# Latent class analysis of sleep-related worry and its influencing factors among shift work nurses: a cross-sectional study

**DOI:** 10.3389/fpubh.2026.1908778

**Published:** 2026-09-03

**Authors:** Shuangli Zhang, Zhilin Cheng, Jianhong Qiao

**Affiliations:** 1 School of Nursing, Shandong Second Medical University, Weifang, Shandong, China; 2 The First Affiliated Hospital of Shandong First Medical University (Shandong Provincial Qianfoshan Hospital), Jinan, Shandong, China; 3 School of Stomatology, Shandong First Medical University, Jinan, Shandong, China

**Keywords:** latent class analysis, nurses, nursing management, shift work, sleep-related worry

## Abstract

**Objective:**

To identify the distinct profiles of sleep-related worry among shift-work nurses and explore their influencing factors, thereby providing a basis for developing targeted interventions for nursing management.

**Methods:**

A convenience sampling method was used to select 653 shift nurses from three tertiary grade A hospitals in Shandong Province from March to July 2025 as the study participants. Data were collected using a general information questionnaire, the Anxiety and Preoccupation about Sleep Questionnaire, the Self-regulatory Fatigue Scale, the Pittsburgh Sleep Quality Index, and the Stanford Presenteeism Scale-6. Latent Class Analysis was employed to identify distinct subgroups of sleep-related worry. Influencing factors across the identified classes were examined using univariate analysis and multinomial logistic regression.

**Results:**

The sleep-related worry score of the 653 shift nurses was (35.27 ± 9.07) points. They could be divided into three classes: the “emotionally healthy” sleep-related worry group (16.54%), the “consequence worry” sleep-related worry group (25.11%), and the “generalized dysregulation” sleep-related worry group (58.35%). The results of multinomial logistic regression analysis showed that sleep quality, self-regulatory fatigue, sleep-related training, average monthly income, and overtime work were influencing factors of the latent classes of sleep-related worry among shift nurses (*p* < 0.05).

**Conclusion:**

Shift-work nurses report a moderate to high level of sleep-related worry, which manifests in heterogeneous profiles. Nursing managers should accurately identify the differential needs of nurses across these distinct worry categories and implement tailored interventions to improve both sleep health and nursing care quality.

## 1 Introduction

Nurses, as an important component of the healthcare system, undertake heavy medical tasks. Shift nurses, working under three-shift or irregular shift schedules, commonly experience sleep-related worry, characterized by excessive concerns about difficulty falling asleep or sleep interruption. Sleep-related worry can disrupt shift nurses’ physiological rest at night and extend into daytime work, leading to attention deficits and emotional fluctuations. It has become a significant threat to nurses’ occupational health and patient safety.

Sleep-related worry refers to a psychological state characterized by concerns about the consequences of poor sleep and diminished perceived control over sleep, as well as worry and anxiety about sleep quality and sleep problems (1). Jansson-Fröjmark conceptualized sleep-related worry as a “24-h phenomenon,” meaning that the worry is not limited to the pre-sleep period but extends across the entire circadian cycle (2). Closely related to but qualitatively distinct from sleep-related worry is sleep insufficiency, which specifically refers to sleep duration that is shorter than what is needed to maintain daytime wakefulness. It should be noted that sleep insufficiency and sleep-related worry are two qualitatively distinct phenomena: the former refers to a physiological reduction in sleep duration or quality, whereas the latter denotes a cognitive-emotional process involving negative appraisal of and excessive preoccupation with sleep and its consequences. These two constructs are mutually influential.

According to the hyperarousal model proposed by Riemann et al. (3), chronic sleep insufficiency can disrupt the negative feedback regulation of the hypothalamic–pituitary–adrenal (HPA) axis, leading to dysregulated cortisol secretion rhythm and enhanced amygdala sensitivity to negative stimuli, thereby placing shift nurses in a state of physiological hyperarousal characterized by being “tired but wired.” This hyperaroused state prolongs sleep onset latency and exacerbates nocturnal sleep fragmentation. Sleep insufficiency can lead to cumulative daytime fatigue and impaired emotion regulation. Daytime functional impairment causes shift nurses to become overly preoccupied with sleep problems, thereby triggering sleep-related worry. Sleep-related worry readily activates the sympathetic nervous system and elevates cortisol levels, keeping the body in a hyperaroused state. This hyperaroused state prolongs sleep onset latency and exacerbates sleep fragmentation, further worsening the pre-existing sleep insufficiency among shift nurses. Thus, sleep insufficiency and sleep-related worry mutually reinforce each other through hyperarousal, forming a closed-loop vicious cycle.

The study found that 75.72% of shift nurses had sleep problems, and one-third of nurses had concerns about insufficient sleep (4). Factors such as unreasonable shift scheduling, poor nursing work environment conditions, and insufficient managerial support can reduce nurses’ satisfaction with sleep adequacy and trigger concerns about sleep problems related to shift work (5). Over 80% of nurses reported that continuous work pressure easily induces physiological arousal, and sleep environment disturbances lead to sleep fragmentation, causing persistent concerns about sleep health among shift nurses (6). A cross-sectional study involving 289 shift nurses in the emergency department showed that 57.3% of them experienced sleep-related worry. This phenomenon was particularly prominent among nurses aged ≤25 years and those with <5 years of work experience, whose sleep-related worry scores were significantly higher than those of more senior nurses, and were often accompanied by strong feelings of helplessness, confusion, and long-term anxiety (7). Research in China has shown that sleep-related worry among anesthesiology nurses is at a moderately high level, and the current state of sleep-related worry is not optimistic (8).

Chronic sleep insufficiency and sleep-related worry can significantly increase nurses’ risks of physical and mental health problems, including the incidence of psychological disorders such as anxiety and depression (9, 10), as well as chronic physical conditions such as obesity and cardiovascular disease (11, 12). Severe sleep-related worry can impair nurses’ cognitive function (13, 14), lead to decreased work efficiency (15), reduce the quality of nursing care provided to patients, and affect patient treatment outcomes and safety (16). Nurse–patient communication is hindered, resulting in decreased patient understanding of and cooperation with treatment (17). Due to sleep insufficiency or sleep-related worry, nurses face an increased risk of medical errors and adverse events (18), as well as a higher risk of patient complaints and legal actions. This leads to reduced job satisfaction among nurses, exacerbates the nurse turnover rate (19), drives up recruitment difficulty and labor costs, and forces healthcare institutions to invest more resources to address potential risks.

There are many factors that contribute to sleep-related worry among shift nurses. Shift nurses often face decreased sleep quality, disrupted sleep patterns, and physical and mental health problems caused by sleep disorders (20). Research shows that nurses working 16-h shifts exhibit high levels of cumulative fatigue, and the degree of fatigue is significantly negatively correlated with sleep quality (21). Excessive worry about sleep problems increases autonomic nervous system arousal and emotional distress before sleep (22). Chronic presenteeism continuously consumes nurses’ psychological resources, leading to increased work stress and decreased self-efficacy (23, 24). Psychological energy depletion and self-regulatory fatigue induced by shift work stress weaken nurses’ emotional regulation ability and the spread of negative thinking, thereby exacerbating sleep-related worry (25). 63.7% of shift nurses had experienced presenteeism in the past 4 weeks. Among nurses, insomnia, presenteeism, and nursing omission events were positively correlated with each other, and there was a positive association between presenteeism and persistent sleep-related worry (26, 27).

Current research on sleep-related worry among shift nurses is predominantly variable-centered, focusing on linear associations or causal pathways between sleep-related worry and various influencing factors. This approach treats the study sample as a homogeneous whole, calculating group-level means, correlation coefficients, or regression coefficients to describe “average effects” among variables. It assumes that all individuals follow the same response pattern, thereby overlooking the heterogeneity of the shift nurse population. Latent class analysis (LCA) reveals population heterogeneity by identifying the correlational characteristics among manifest variables (28). LCA accurately identifies the characteristics of each class by maximizing between-group differences and minimizing within-group differences. LCA is a statistical method specifically designed for categorical manifest variables, enabling precise classification of individuals based on their response patterns across items. If ordered categorical data are treated as continuous variables in latent profile analysis (LPA), the resulting estimates may be biased. Therefore, this study employed latent class analysis (LCA) to identify distinct classes of sleep-related worry among shift nurses, explored the effects of sociodemographic characteristics, presenteeism, sleep quality, and self-regulatory fatigue on sleep-related worry, and examined the influencing factors across different classes. The findings are expected to provide a reference for nursing managers to develop personalized intervention programs and improve nurses’ sleep quality.

## 2 Materials and methods

### 2.1 Study design

It was a cross-sectional study.

### 2.2 Study population

A convenience sampling method was used to select shift nurses working on the frontlines of three tertiary grade A hospitals in Shandong Province from March to July 2025. Inclusion criteria: (1) frontline clinical nurses holding a valid nursing practice certificate; (2) continuous participation in shift work for the past 6 months; (3) provided informed consent and voluntarily participated in the study. Exclusion criteria:(1) intern, refresher, or standardized training nurses not employed by the hospital; (2) nurses on sick leave, personal leave, maternity leave, or other types of absence; (3) nursing staff working in auxiliary departments such as supply rooms, hospital pharmacies, medical imaging, and clinical laboratories.

**Figure 1:**
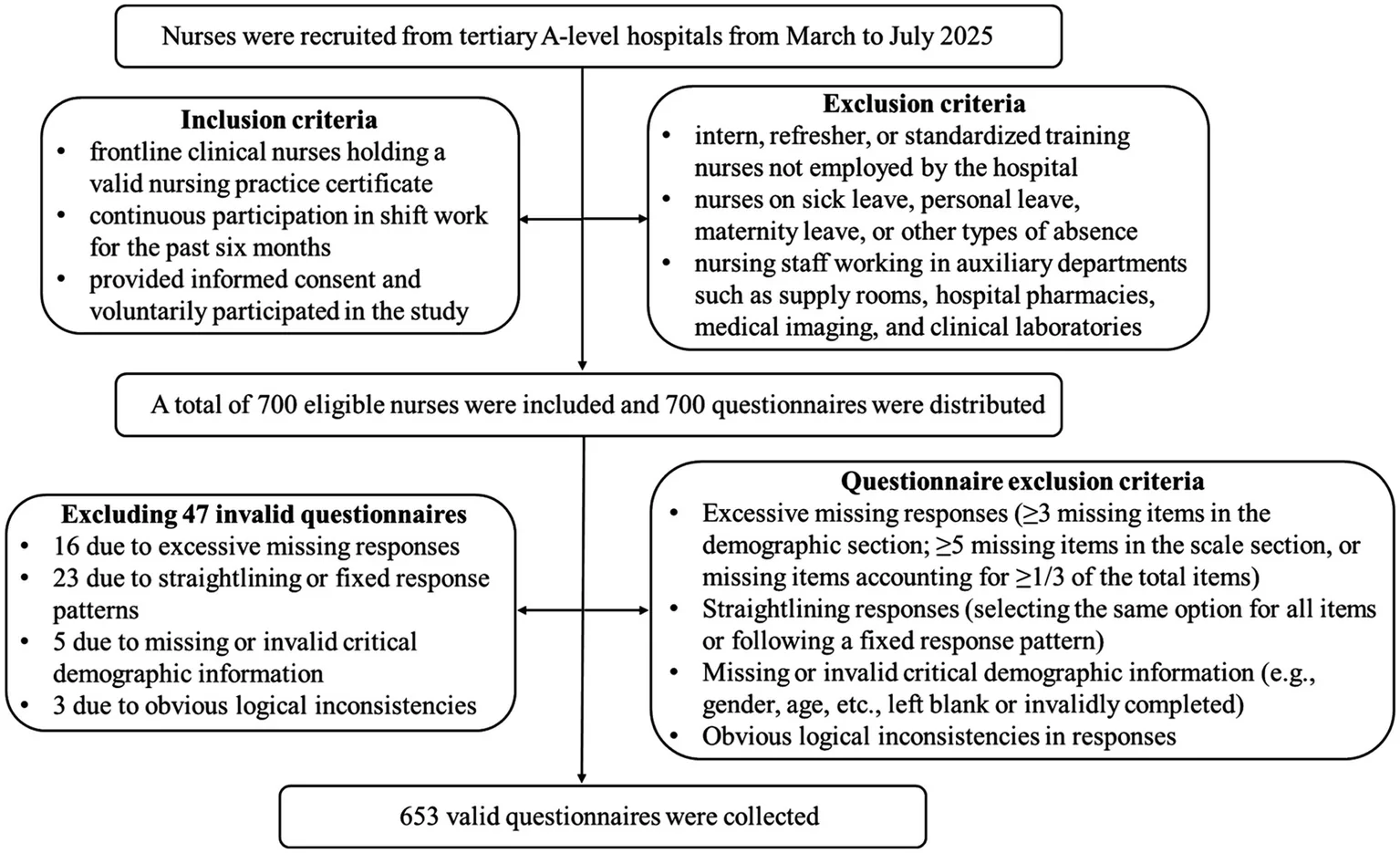
Participant recruitment flowchart.

According to Kendall’s sample size calculation principle, the sample size should be 5 to 10 times the number of independent variable (29). A total of 26 variables were included in this study. According to the recommended sample size of 5–10 times the number of variables, the required sample size was 130–260 cases. Considering a 20% attrition rate due to incomplete or invalid questionnaires, the final required sample size was calculated to be 156–312 cases. A total of 700 questionnaires were distributed. After excluding 47 invalid questionnaires (16 due to excessive missing responses, 23 due to straightlining or fixed response patterns, 5 due to missing or invalid critical demographic information, and 3 due to obvious logical inconsistencies), 653 valid questionnaires were obtained, yielding an effective response rate of 93.3%. A flowchart of participant recruitment is illustrated in Figure 1.

### 2.3 Research instruments

#### 2.3.1 General information questionnaire

This study used a self-designed general information questionnaire to collect demographic characteristics and shift work-related characteristics. Demographic characteristics: age, gender, marital status, education level, years of work experience, average monthly income, self-rated health status, self-rated sleep status, sleep-related training, mental health training. Shift work characteristics: shift pattern, shift length (12 h/8 h), shift rotation direction (clockwise/counterclockwise), frequency of working more than 40 h per week.

#### 2.3.2 Anxiety and preoccupation about sleep questionnaire

The APSQ was developed by Jansson-Fröjmark et al. (2) and revised by Shi Xuliang (10), was used to assess the level of sleep-related worry among shift nurses. The ASPQ consists of two dimensions: worry about the consequences of poor sleep (6 items) and worry about the uncontrollability of sleep (4 items), with a total of 10 items. A Likert 5-point scale was used, ranging from 1 (“strongly disagree”) to 5 (“strongly agree”). Total scores ranged from 10 to 50, with higher scores indicating greater sleep-related worry. In this study, the *Cronbach’s α* coefficient of the scale was 0.932.

#### 2.3.3 Self-regulatory fatigue scale

The SRF-S was developed by Nes (30) and translated and revised by Ligang W (31)to assess patients’ level of self-regulatory fatigue. The SRF-S consists of three dimensions (cognitive control, emotional control, and behavioral control), with a total of 16 items. The *Cronbach’s α* coefficient of the scale was 0.840. A Likert 5-point scale was used, ranging from 1 (“strongly disagree”) to 5 (“strongly agree”). Total scores ranged from 16 to 80, with higher scores indicating more severe self-regulatory fatigue. In this study, the *Cronbach’s α* coefficient of the scale was 0.842.

#### 2.3.4 Pittsburgh sleep quality index

The PSQI was developed by Buysse (32) to assess participants’ sleep quality over the past month. The PSQI consists of seven dimensions (subjective sleep quality, sleep latency, actual sleep duration, sleep efficiency, sleep disturbances, use of hypnotic medications, and daytime dysfunction), with a total of 18 items. The *Cronbach’s α* coefficient is 0.85, indicating high reliability and validity. The scale consists of 4 fill-in-the-blank questions and 14 multiple-choice questions. Each dimension is scored from 0 to 3 based on the participant’s response. The total PSQI score ranges from 0 to 21, with higher scores indicating poorer sleep quality. A total PSQI score of ≥7 indicates the presence of sleep disturbance. In this study, the *Cronbach’s α* coefficient of the scale was 0.726.

#### 2.3.5 Stanford presenteeism scale-6

The SPS-6 was developed by Koopman (33) and adapted into Chinese by Zhao Fang (34). It has been used to measure presenteeism among nurses in China. The SPS-6 consists of two dimensions (work limitation and work energy), with a total of 6 items. The *Cronbach’s α* coefficient is 0.93. A Likert 5-point scale was used, ranging from 1 (“strongly disagree”) to 5 (“strongly agree”). Total scores ranged from 6 to 30, with higher scores indicating greater productivity loss due to presenteeism. In this study, the *Cronbach’s α* coefficient of the scale was 0.730.

### 2.4 Data collection and quality control methods

A paper-based survey was conducted on-site, and permission for site access was obtained from the nursing departments and head nurses of each participating hospital. The researcher personally visited each department of the participating hospitals. Survey sessions were scheduled during non-peak work hours (e.g., after shift handovers or before professional training sessions), and temporary survey stations were set up in separate conference rooms or rest areas within each department. Using standardized instructions, the researcher provided a collective explanation to all nurses on duty that day regarding the study purpose, content, estimated completion time (approximately 20–30 min), and the voluntary nature of participation. The researcher emphasized both orally and in writing that participation in the study, as well as the decision to complete all items, was entirely voluntary and bore no relation to participants’ performance appraisals, shift scheduling, leave arrangements, career advancement, or any working relationships with head nurses or administrative management. Nurses who declined to participate or withdrew from the study at any time were not required to provide any reason, and such decisions would not lead to any adverse consequences of any kind. Following the completion of informed consent, the researcher administered the paper questionnaires directly to the nurses. The questionnaire did not include any items that could identify individual respondents. For the sole purpose of internal page verification, a non-sequential random verification code was pre-generated by the study’s backend system and printed on the lower right corner of the final page. This code bore no correspondence to the order of distribution, the department, or any specific participant. Throughout the questionnaire completion process, nurses filled out the questionnaires independently and by themselves. The researcher remained at the entrance of the survey station and only approached the participants when they raised their hands to ask objective questions about the wording of specific items. Upon completion, nurses sealed the questionnaires themselves and placed them into an opaque ballot box. Throughout the entire collection process, neither the researcher, head nurses, nor any third party could link any specific questionnaire to its respondent. After questionnaire collection, all returned questionnaires were unboxed collectively and screened according to the predefined exclusion criteria. Data entry was then performed independently by two researchers. Prior to data entry, all valid questionnaires were recoded to remove any original identifying information. Following data entry, the two independently entered datasets were cross-checked bidirectionally. In the event of inconsistencies, the original questionnaires were reviewed for correction. The amendment procedure was carried out without reference to any personally identifiable information. Ultimately, the final dataset contained only study-assigned identification codes, and all subsequent analyses were conducted on the pooled aggregate data. As a token of appreciation for their cooperation, each nurse who attended the survey session received a small gift placed at their seat before the questionnaire began, regardless of whether they eventually completed the questionnaire or withdrew midway. The gifts were distributed independently by the researcher, without involvement from head nurses or administrative staff, and were not contingent upon completion of all questionnaire items. To ensure the target sample size, the study planned to collect 600 valid questionnaires. A total of 700 questionnaires were distributed, and 653 valid responses were obtained.

### 2.5 Statistical analysis

Statistical analyses were performed using SPSS 27.0 and Mplus 8.0 software. Frequencies and percentages were used to describe the general demographic information and the scores of the APSQ, SRF-S, PSQI, and SPS-6 among shift nurses. Chi-square tests and analysis of variance (ANOVA) were used to test the differences in characteristics among different latent groups of sleep-related worry in shift nurses. Factors with statistically significant differences were entered into logistic regression analysis for testing. Data from the APSQ were dichotomized into 0 or 1: if the average score of items in each dimension was ≥3, it was converted to 1; otherwise, it was converted to 0. LCA of sleep-related worry among shift nurses was conducted using Mplus 8.3 software, starting with a one-class model and progressively increasing the number of classes to determine the best-fitting model. Model fit was evaluated using the Akaike information criterion (AIC), Bayesian information criterion (BIC), sample-size-adjusted BIC (aBIC), entropy, the bootstrapped likelihood ratio test (BLRT), and the Lo–Mendell–Rubin likelihood ratio test (LMRT) to select the most appropriate model. Among these, smaller values of AIC, BIC, and a BIC indicate better model fit; entropy values closer to 1 indicate more accurate classification; and *p* < 0.05 for BLRT and LMRT indicates that the k-class model fits significantly better than the (k-1)-class model. All statistical tests were two-tailed, with a significance level set at *p* < 0.05.

## 3 Results

### 3.1 Characteristics of emergency department nurses

Among the 653 shift nurses, there were 114 males (17.6%) and 539 females (82.4%). The majority held a bachelor’s degree (90.5%). Regarding employment status, contract-based employees accounted for the largest proportion (70.6%). Clockwise shift rotation was predominant (425, 65.1%). Other general demographic characteristics are presented in Table 1.

**Table 1:** Sociodemographic characteristics of research subjects and inter group comparisons.

Characteristics	Category (assignment)	Total(*n* = 653)	Classification of latent profiles			χ^2^	*p*
			C1 (*n* = 108)	C2 (*n* = 164)	C3 (*n* = 381)		
Gender	Male (0)	114(17.5)	17(15.7)	27(16.5)	70(18.4)	0.758	0.772
	Female (1)	539(82.5)	91(84.3)	137(83.5)	311(81.6)		
Age (years)	≤30 (0)	297(45.5)	38(35.2)	88(53.7)	171(44.9)	9.153	0.057
	31–40 (1)	298(45.6)	58(53.7)	64(39.0)	176(46.2)		
	>40 (2)	58(8.9)	12(11.1)	12(7.3)	34(8.9)		
Marital status	Unmarried (0)	197(30.2)	27(25.0) ^a^	65(39.6) ^a^	105(27.6) ^a,b^	29.661	<0.001
	Married (1)	442(67.7)	71(65.7) ^a^	99(60.4) ^b^	272(71.4) ^b^		
	Other (2)	14(2.1)	10(9.3) ^b^	0(0.0) ^b^	4(2.1) ^a^		
Education level	Technical secondary school (0)	7(1.1)	0(0.0)	2(1.2)	5(1.3)	1.907	0.941
	Associate degree (1)	24(3.7)	3(2.8)	7(4.3)	14(3.7)		
	Bachelor’s degree (2)	591(90.5)	101(93.5)	146(89.0)	344(90.3)		
	Master’s degree or above (3)	31(4.7)	4(3.7)	9(5.5)	18(4.7)		
Years of work experience	0–1(0)	30(4.6)	8(7.4) ^a^	11(6.7) ^a^	11(2.9) ^a^	15.653	0.048
	2–5(1)	176(27.0)	26(24.1) ^a, b^	57(34.8) ^b^	93(24.4) ^a^		
	6–10(2)	237(36.3)	37(34.3) ^a^	55(33.5) ^a^	145(38.1) ^a^		
	11–15(3)	136(20.8)	23(21.3) ^a^	25(15.2) ^a^	88(23.1) ^a^		
	≥16 (4)	74(11.3)	14(13.0) ^a^	16(9.8) ^a^	44(11.5) ^a^		
Monthly income (RMB)	<4,000(0)	15(2.3)	5(4.6) ^a^	4(2.4) ^a^	6(1.6) ^a^	18.175	0.020
	4,000–6,000(1)	66(10.1)	19(17.6) ^a^	19(11.6) ^a, b^	28(7.3) ^b^		
	6,001–8,000(2)	155(23.7)	29(26.9) ^a^	37(22.6) ^a^	89(23.4) ^a^		
	8,001–10,000(3)	271(41.5)	32(29.6) ^a^	67(40.9) ^a, b^	172(45.1) ^b^		
	>10,000(4)	146(22.4)	23(21.3) ^a^	37(22.6) ^a^	86(22.6) ^a^		
Self-rated sleep quality	Excellent (0)	54(8.3)	30(27.8) ^a^	16(9.8) ^b^	8(2.1) ^c^	179.492	<0.001
	Good (1)	166(25.4)	52(48.1) ^a^	56(34.1) ^a^	58(15.2) ^b^		
	Fair (2)	301(46.1)	23(21.3) ^a^	80(48.8) ^b^	198(52.0) ^b^		
	Poor (3)	107(16.4)	2(1.9) ^a^	12(7.3) ^a^	93(24.4) ^b^		
	Very poor (4)	25(3.8)	1(0.9) ^a, b^	0(0.0) ^b^	24(6.3) ^a^		
Self-rated health status	Excellent (0)	56(8.6)	28(25.9) ^a^	14(8.5) ^b^	14(3.7) ^b^	117.822	<0.001
	Good (1)	256(39.2)	60(55.6) ^a^	83(50.6) ^a^	113(29.7) ^b^		
	Fair (2)	294(45.0)	19(17.6) ^a^	61(37.8) ^b^	213(55.9) ^c^		
	Poor (3)	39(6.0)	0(0.0) ^a^	5(3.0) ^a^	34(8.9) ^b^		
	Very poor (4)	8(1.2)	1(0.9) ^a^	0(0.0) ^a^	7(1.8) ^a^		
Sleep-related training	frequently (0)	36(5.5)	24(22.2) ^a^	3(1.8) ^b^	9(2.4) ^b^	107.196	<0.001
	Sometimes (1)	74(11.3)	28(25.9) ^a^	16(9.8) ^b^	30(7.9) ^b^		
	Occasionally (2)	102(15.6)	8(7.4) ^a^	24(14.6) ^a, b^	70(18.4) ^b^		
	Never (3)	441(67.5)	48(44.4) ^a^	121(73.8) ^b^	272(71.4) ^b^		
Mental health training	frequently (0)	40(6.1)	22(20.4) ^a^	5(3.0) ^b^	13(3.4) ^b^	67.903	<0.001
	Sometimes (1)	74(11.3)	29(26.9) ^a^	29(17.7) ^a, b^	46(12.1) ^b^		
	Occasionally (2)	102(15.6)	22(20.4) ^a^	39(23.8) ^a^	89(23.4) ^a^		
	Never (3)	441(67.5)	35(32.4) ^a^	91(55.5) ^b^	233(61.2) ^b^		
Shift rotation direction	Clockwise (0)	499(76.4)	76(70.4)	133(81.1)	290(76.1)	4.204	0.122
	Counterclockwise (1)	154(23.6)	32(29.6)	31(18.9)	91(23.9)		
Shift pattern	Two-shift system (0)	261(40.0)	27(25.0) ^a^	77(47.0) ^b^	157(41.2) ^b^	17.200	0.002
	Three-shift system (1)	366(56.0)	79(73.1) ^a^	81(49.4) ^b^	206(54.1) ^b^		
	Irregular (2)	26(4.0)	2(1.9) ^a^	6(3.7) ^a^	18(4.7) ^a^		
Shift length	12-h shift (0)	113(17.3)	10(9.3) ^a^	22(13.4) ^a, b^	81(21.3) ^b^	10.784	0.005
	8-h shift (1)	540(82.7)	98(90.7) ^a^	142(86.6) ^a, b^	300(78.7) ^b^		
Frequency of working more than 40 h per month	1 (0)	193(29.6)	29(26.9) ^a^	60(36.6) ^a^	104(27.3) ^a^	22.267	0.001
	2 (1)	149(22.8)	31(28.7) ^a^	42(25.6) ^a^	76(19.9) ^a^		
	3 (2)	121(18.5)	28(25.9) ^a^	18(11.0) ^b^	75(19.7) ^a^		
	4 (3)	190(29.1)	20(18.5) ^a^	44(26.8) ^a, b^	126(33.1) ^b^		

C1, emotionally healthy sleep-related worry group, C2, consequence worry sleep-related worry group, C3, generalized dysregulation sleep-related worry group. *Post-hoc* multiple comparisons were performed using the column proportion Z-test, with the significance level set at α = 0.05. Within each categorical variable, different subscript letters (a, b, c) indicate statistically significant differences in column proportions between latent classes; groups sharing the same letter do not differ significantly.

### 3.2 Results of latent class analysis of sleep-related worry among shift nurses

The fitting metrics were displayed in Table 2. The 10 items of the APSQ were entered as manifest variables in the latent class analysis. Starting from a one-class model, the number of classes was gradually increased, and a total of five models were fitted. As the number of classes increased, the values of AIC, BIC, and aBIC gradually decreased. When five latent classes were retained, the *p*-value of the LMRT was >0.05, indicating that the model was not statistically significant. For Model 3, the entropy was 0.903, and the *p*-values of both the LMRT and BLRT were <0.05. The results of the LMRT and BLRT for Model 4 were also statistically significant. However, based on the practical interpretability of the model and the sample sizes of the latent classes, Model 3 was ultimately selected in this study, with class probabilities of 0.5835, 0.2511, and 0.1654.

**Table 2:** Fit statistics for each profile structure.

Model	*AIC*	*BIC*	*aBIC*	*Entropy*	*LMRT*	*BLRT*	Probability of class
1-Class	6867.586	6912.402	6880.652	-	-	-	100
2-Class	4883.455	4977.568	4910.893	0.913	<0.001	<0.001	65.54/34.46
3-Class	4547.044	4690.455	4588.855	0.904	<0.001	<0.001	16.54/25.11/58.35
4-Class	4506.471	4699.179	4562.654	0.887	0.039	<0.001	54.53/16.38/8.73/20.36
5-Class	4483.863	4725.868	4554.418	0.853	0.227	<0.001	8.88/54.06/11.18/11.64/14.24

To evaluate the classification accuracy and reliability of the latent class analysis results, the average probabilities of the three classes were calculated. The results showed that the average probabilities of correctly classifying samples into their own classes were 95.0, 93.0, and 97.6%, respectively, all above the recommended threshold of 80%, indicating that the three-class model had high classification quality and the results were reliable. The membership probabilities for the three latent classes are shown in Table 3.

**Table 3:** Average attribution probabilities for each latent profile.

	Class1(%)	Class2(%)	Class3(%)
Class1	0.950	0.050	0.000
Class2	0.030	0.930	0.039
Class3	0.000	0.024	0.976

C1, emotionally healthy sleep-related worry group, C2, consequence worry sleep-related worry group, C3, generalized dysregulation sleep-related worry group.

### 3.3 Latent class analysis of sleep-related worry among shift nurses

**Figure 2:**
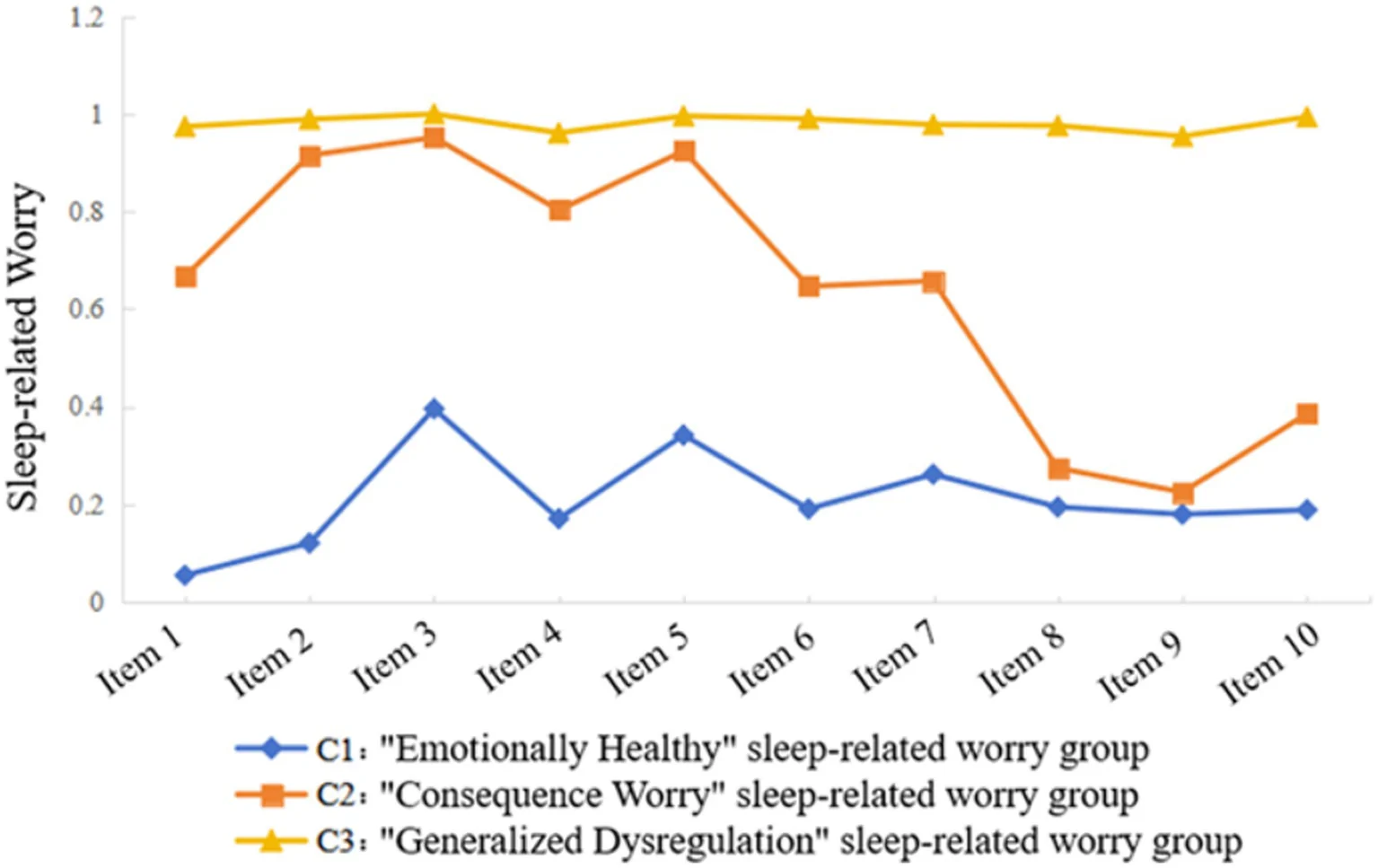
Latent class characteristics of sleep-related worry among shift nurses.

Based on the conditional probability distributions of the items, the characteristic attributes of each class were named, as shown in Figure 1. Shift nurses in C1 group had only mild worry about the potential health impacts and mood swings caused by sleep; therefore, this class was named the “Emotionally Healthy” sleep-related worry group, accounting for 16.54% (108 cases). C2 group shift nurses had a high level of worry about the negative effects of sleep insufficiency on health, mood, work, and social interactions, but their level of worry about the sleep process itself was moderate. This indicates that their worry focused on the “consequences” rather than the “process” of sleep; therefore, this class was named the “Consequence Worry” sleep-related worry group, accounting for 25.11% (164 cases). For C3 group, worry had spread from sleep to multiple dimensions, manifesting as generalized dysregulation of sleep-related worry. Therefore, this class was named the “Generalized Dysregulation” sleep-related worry group, accounting for 58.35% (381 cases; Figure 2).

### 3.4 Univariate analysis of latent classes of sleep-related worry among shift nurses

The univariate analysis results showed that different classes of sleep-related worry among shift nurses had statistically significant differences in demographic characteristics: marital status (*χ^2^* = 29.661, *p* < 0.001), years of work experience (*χ^2^* = 15.653, *p* = 0.048), average monthly income (*χ^2^* = 18.175, *p* = 0.020), self-rated sleep quality (*χ^2^* = 179.492, *p* < 0.001), self-rated health status (*χ^2^* = 117.822, *p* < 0.001), sleep-related training (*χ^2^* = 107.196, *p* < 0.001), and mental health training (*χ^2^* = 67.903, *p* < 0.001). Different classes of sleep-related worry among shift nurses showed statistically significant differences in shift work characteristics: shift pattern (*χ^2^* = 17.200, *p* = 0.002), shift length (*χ^2^* = 10.784, *p* = 0.005), and frequency of working more than 40 h per month (*χ^2^* = 22.267, *p* = 0.001). Post-hoc comparisons showed that the proportion of married nurses in C3 group was significantly higher than that in C2 group. For nurses with 2–5 years of work experience, the proportion in C2 group was significantly higher than that in C3 group. In addition, the proportion of nurses with a monthly income below 6,000 RMB was significantly higher in C1 group than in C3 group. In C1 group, the proportion of nurses reporting “excellent/good” sleep quality was significantly lower than that in C2 group and C3 group, whereas the proportion reporting “poor/very poor” sleep quality was significantly higher than in the other two classes. A similar pattern was observed for self-rated health status, with C3 group having the highest proportion of “good/excellent” ratings and C1 group having the lowest. The proportion of nurses in C1 group who “frequently” participated in sleep-related training and mental health training was significantly higher than that in C2 group and C3 group (all *p* < 0.001), which may reflect the high demand for health resources in this group, as shown in Table 1.

### 3.5 Multivariable analysis of latent classes of sleep-related worry among shift nurses

Using the three latent classes of sleep-related worry identified by latent class analysis as the dependent variable, with the “Emotionally Healthy” sleep-related worry group as the reference category, we entered the indicators that showed statistically significant differences in the univariate analysis as independent variables into a multinomial logistic regression. The assignment of independent variables is presented in Table 1. The following variables were entered as independent variables into the multinomial logistic regression model using the Enter method (forced entry): sleep quality, self-regulatory fatigue, presenteeism, years of work experience, average monthly income, sleep-related training, mental health training, shift pattern, shift length, whether weekly working hours exceeded 40 h, as well as the recoded self-rated sleep status, self-rated health status, and marital status. The recoded variables were assigned as follows: marital status: unmarried (0) versus married/divorced (1); self-rated sleep quality: good (0) versus poor (1); self-rated health status: good (0) versus poor (1). The results showed that, compared with the “Emotionally Healthy” sleep-related worry group, higher levels of poor sleep quality (*OR* = 2.470, 9*5%C*I: 2.078–2.936), higher self-regulatory fatigue (*OR* = 1.145, *95%CI*: 1.076–1.219), and higher presenteeism (*OR* = 1.114, *95%CI*: 1.008–1.232) were associated with an increased risk of being classified into the “Generalized Dysregulation” sleep-related worry group. In addition, using a monthly income >10,000 RMB as the reference, nurses with a monthly income of 6,001–8,000 RMB (*OR* = 6.717, *95%CI*: 1.993–22.639) had a higher risk. Using “never participated” as the reference, nurses who “occasionally” participated in sleep-related training (*OR* = 5.363, *95%CI*: 1.287–22.353) had a higher risk. Using “overtime ≥4 times per month” as the reference, nurses who worked overtime 3 times per month (*OR* = 3.722, *95%CI*: 1.101–12.591) had a higher risk of being in the “Generalized Dysregulation” group. Compared with the “Emotionally Healthy” sleep-related worry group, poorer sleep quality (*OR* = 1.579, *95%CI*: 1.361–1.832), higher self-regulatory fatigue (OR = 1.084, *95%CI*: 1.023–1.148), and working overtime once per month (*OR* = 2.659, *95%CI*: 1.024–6.902) were significantly associated with an increased risk of being classified into the “Consequence Worry” sleep-related worry group. Nurses who “frequently” participated in sleep-related training had a significantly lower risk of being classified into the “Consequence Worry” sleep-related worry group (*OR* = 0.135, *95%CI*: 0.018–0.988), suggesting that frequent participation in sleep-related training is a protective factor against membership in this class, as shown in Table 4.

**Table 4:** Multinomial logistic regression analysis of latent classes of sleep-related worry among shift nurses (*n* = 653).

Variables	C2 vs. C1					C3 vs. C1				
	*β*	*SE*	*Waldχ^2^*	*p*	*OR (95% CI)*	*β*	*SE*	*Waldχ^2^*	*p*	*OR (95% CI)*
Marital status										
Unmarried	0.309	0.567	0.297	0.586	1.362(0.448–4.143)	0.184	0.740	0.062	0.804	1.202(0.282–5.122)
Self-rated sleep quality										
Good sleep quality	0.994	0.757	1.727	0.189	2.703(0.613–11.913)	−1.949	1.394	1.955	0.162	0.142(0.009–2.188)
Self-rated health status										
Good self‑rated health	−1.936	1.346	2.069	0.150	0.144(0.010–2.018)	0.132	0.619	0.045	0.832	1.141(0.339–3.837)
Years of work experience										
0-1(years)	−0.628	1.046	0.360	0.549	0.534(0.069–4.149)	−1.778	1.223	2.111	0.146	0.169(0.015–1.859)
2-5(years)	0.065	0.732	0.008	0.929	1.067(0.254–4.483)	−0.296	0.841	0.124	0.725	0.744(0.143–3.863)
6-10(years)	−0.798	0.601	1.763	0.184	0.450(0.139–1.462)	−0.759	0.696	1.188	0.276	0.468(0.120–1.833)
11-15(years)	−0.831	0.649	1.637	0.201	0.436(0.122–1.566)	−0.594	0.746	0.633	0.426	0.552(0.128–2.384)
Monthly income (RMB)										
<4,000	0.624	1.118	0.311	0.577	1.866(0.209–16.696)	2.009	1.325	2.297	0.130	7.454(0.555–100.112)
4,000–6,000	0.654	0.691	0.896	0.344	1.923(0.496–7.451)	1.244	0.780	2.541	0.111	3.468(0.752–16.004)
6,001–8,000	0.899	0.564	2.539	0.111	2.457(0.813–7.420)	1.905	0.620	9.440	0.002^*^	6.717(1.993–22.639)
8,001–10,000	0.641	0.467	1.884	0.170	1.897(0.760–4.735)	1.001	0.517	3.748	0.053	2.72(0.988–7.493)
Sleep-related training										
Frequently	−2.005	1.017	3.886	0.049^*^	0.135(0.018–0.988)	−1.718	1.233	1.941	0.164	0.179(0.016–2.011)
Sometimes	−0.625	0.720	0.753	0.385	0.535(0.131–2.195)	0.455	0.806	0.319	0.572	1.577(0.325–73,659)
Occasionally	0.819	0.677	1.463	0.226	2.267(0.602–8.543)	1.680	0.728	5.319	0.021^*^	5.363(1.287–22,353)
Mental health training										
Frequently	−1.190	0.910	1.708	0.191	0.304(0.051–1.812)	−1.239	1.082	1.311	0.252	0.290(0.035–2.416)
Sometimes	−0.019	0.721	0.001	0.979	0.981(0.239–4.030)	−0.614	0.785	0.613	0.434	0.541(0.116–2.518)
Occasionally	−0.120	0.467	0.066	0.797	0.887(0.355–2.214)	−0.433	0.526	0.679	0.410	0.648(0.231–1.817)
Shift length										
12‑hour shift	−0.022	0.572	0.002	0.969	0.978(0.319–3.001)	0.171	1.198	0.020	0.886	1.187(0.113–12.424)
Shift pattern										
Two-shift system	0.185	1.128	0.027	0.870	1.203(0.132–10.983)	−0.265	1.171	0.051	0.821	0.767(0.077–7.618)
Three-shift system	−0.518	1.104	0.220	0.639	0.596(0.069–5.181)	0.474	0.625	0.574	0.449	1.606(0.471–5.471)
Frequency of working more than 40 h per month										
1	0.978	0.487	4.036	0.045^*^	2.659(1.024–6.902)	0.937	0.529	3.134	0.077	2.551(0.905–7.197)
2	0.412	0.529	0.608	0.435	1.510(0.536–4.257)	0.217	0.570	0.144	0.704	1.242(0.406–3.795)
3	0.389	0.578	0.454	0.500	1.476(0.476–4.579)	1.314	0.622	4.469	0.035^*^	3.722(1.101–12.591)
Sleep quality	0.457	0.076	36.256	<0.001^*^	1.579(1.361–1.832)	0.904	0.088	105.248	<0.001^*^	2.470 (2.078–2.936)
Self-regulatory fatigue	0.081	0.029	7.487	0.006^*^	1.084(1.023–1.148)	0.136	0.032	18.305	<0.001^*^	1.145(1.076–1.219)
Presenteeism	0.048	0.047	1.057	0.304	1.049(0.957–1.150)	0.108	0.051	4.479	0.034^*^	1.114(1.008–1.232)

C1, emotionally healthy sleep‑related worry group; C2, consequence worry sleep‑related worry group; C3, generalized dysregulation sleep‑related worry group. *The difference was statistically significant (*P*<0.05).

### 3.6 Comparison of sleep-related worry classes with self-regulatory fatigue, sleep quality, and presenteeism scores among shift nurses

In terms of sleep quality, the mean scores across the three classes showed a stepwise increasing trend (C3 > C2 > C1), with values of (5.63 ± 2.82), (9.28 ± 2.70), and (13.08 ± 2.80), respectively. Bonferroni *post-hoc* comparisons revealed that the differences between any two groups were all statistically significant (all *p* < 0.001), indicating that C3 group had the poorest sleep quality, while C1 group had the best. In terms of self-regulatory fatigue, the mean scores across the three classes also exhibited a stepwise increasing trend (C3 > C2 > C1), with values of (36.70 ± 6.41), (43.03 ± 6.97), and (48.25 ± 7.61), respectively. Bonferroni post-hoc comparisons showed that all pairwise differences were statistically significant (all *p* < 0.001), indicating that Class 3 had the highest level of self-regulatory fatigue, while C1 group had the lowest. In terms of presenteeism, the mean scores across the three classes similarly exhibited a stepwise increasing trend (C3 > C2 > C1), with values of (11.44 ± 3.39), (14.23 ± 3.99), and (16.35 ± 4.07), respectively. Bonferroni post-hoc comparisons showed that all pairwise differences were statistically significant (all *p* < 0.001), indicating that presenteeism was most severe in C3 group and least severe in C1 group. Taken together, these findings suggest that C3 group had the poorest sleep quality, the highest level of self-regulatory fatigue, and the most severe presenteeism, whereas C1 group showed the opposite pattern, as shown in Table 5.

**Table 5:** Comparison of sleep-related worry classes with sleep quality, self-regulatory fatigue and presenteeism scores among shift nurses (*n* = 653).

Characteristics	Total scores	Classification of latent profiles			*F*	*p*	Comparison between groups
		C1 (*n* = 108)	C2 (*n* = 164)	C3 (*n* = 381)			
sleep quality	10.90 ± 3.97	5.63 ± 2.82	9.28 ± 2.70	13.08 ± 2.80	340.297	<0.001	C3>C2>C1
self-regulatory fatigue	45.03 ± 8.44	36.70 ± 6.41	43.03 ± 6.97	48.25 ± 7.61	114.622	<0.001	C3>C2>C1
presenteeism	15.01 ± 4.34	11.44 ± 3.39	14.23 ± 3.99	16.35 ± 4.07	272.743	<0.001	C3>C2>C1

C1, emotionally healthy sleep-related worry group, C2, consequence worry sleep-related worry group, C3, generalized dysregulation sleep-related worry group. The pattern C3 > C2 > C1 indicates a decreasing trend in mean scores across the three groups, with C3 having the highest scores, C2 intermediate, and C1 the lowest. Sleep quality (PSQI), self-regulatory fatigue (SRF-S), and presenteeism (SPS-6), are all positively scored. Bonferroni *post-hoc* comparisons showed that the differences between any two groups were statistically significant (all *p* < 0.001). The significance level after Bonferroni *post-hoc* correction was set at α’ = 0.0167.

## 4 Discussion

### 4.1 Sleep-related worry among shift nurses is heterogeneous

The results of this study showed significant heterogeneity in the level of sleep-related worry among shift nurses. Three latent classes were identified: the “Generalized Dysregulation” sleep-related worry group, the “Consequence Worry” sleep-related worry group, and the “Emotionally Healthy” sleep-related worry group. The “Generalized Dysregulation” sleep-related worry group accounted for 58.35% of the sample, showing high endorsement on all items of the sleep-related worry scale. Their worry was characterized by universality, multidimensionality, and high intrusiveness, making this subgroup the largest and most severely impaired high-risk subgroup among the three classes. The “Generalized Dysregulation” sleep-related worry group was predominantly composed of shift nurses who were married, had a moderate monthly income (6,001–8,000 RMB), and frequently worked overtime (3 times/month). The “Consequence Worry” sleep-related worry group accounted for 25.11% of the sample and was composed of shift nurses who were unmarried, occasionally worked overtime (once per month), and had “never participated” in sleep-related training. The “Emotionally Healthy” sleep-related worry group accounted for 16.54% of the sample and was identified as the low-risk subgroup in this study. A monthly income below 4,000 RMB and rarely working overtime were typical characteristics of this group. The disruption of nurses’ circadian rhythms by shift work is a significant trigger for sleep-related worry, with approximately 20–30% of shift workers at risk of shift work sleep disorder (35). Alterations in the light–dark cycle lead to disrupted melatonin secretion rhythms, which in turn impair the normal circadian regulation of the sleep–wake cycle, resulting in a series of problems including difficulty initiating sleep, difficulty maintaining sleep, and excessive daytime sleepiness (36). In this study, only 16.54% of the nurses were able to maintain an “Emotionally Healthy” sleep-related worry profile, suggesting that the current sleep health protection mechanisms under the existing shift work system are far from sufficient to meet the sleep health needs of the vast majority of shift nurses. Sleep-related worry is not merely an occasional concern for a few individuals, but a widely prevalent occupational health issue among shift nurses. These findings provide empirical support for nursing managers to re-evaluate shift scheduling, night shift frequency, work hour management, and the allocation of mental health support systems.

### 4.2 Factors influencing latent classes of sleep-related worry among shift nurses

The present study found that average monthly income, sleep-related training, overtime work, sleep quality, self-regulatory fatigue, and presenteeism were influencing factors of different latent classes of sleep-related worry among shift nurses.

According to the cognitive model of insomnia, individuals may develop catastrophic thinking and excessive worry about the consequences of sleep insufficiency (37). In shift nurses with “Consequence Worry,” sleep-related worry primarily manifests as catastrophic expectations about specific consequences—such as “making mistakes tomorrow due to insufficient sleep”—and has a clear situational focus. As a chronic stressor, persistent poor sleep quality can trigger negative cognitive appraisals of sleep in individuals, leading them to feel a loss of control over their current sleep situation and to fundamentally question their own ability to regulate sleep. Research has shown that sleep-related worry is a consequence of poor sleep quality, and it further impairs sleep by persistently activating negative cognitive-emotional processing (38). Law et al. (39) found that worry levels were positively correlated with prolonged sleep onset latency, decreased sleep quality, and increased morning lingering time. Shorter daytime total sleep duration and poorer sleep quality predicted higher levels of worry the following day. Poor sleep quality triggers excessive worry about sleep, and sleep-related worry is more likely to transition from the “Consequence Worry” type to the “Generalized Dysregulation” type.

Multiple studies have confirmed that self-regulatory fatigue is positively correlated with sleep-related worry (8, 40). The higher the level of self-regulatory fatigue, the more severe the depletion of an individual’s psychological resources, and the worse the ability to cope with sleep problems. Due to frequent circadian rhythm shifts, high work demands, and complex interpersonal interactions, shift nurses must continuously mobilize self-regulatory resources to maintain professional efficacy and emotional stability. Long-term, high-intensity psychological resource consumption makes shift nurses a high-risk population for self-regulatory fatigue (25). Self-regulatory fatigue weakens shift nurses’ ability to cope with stress and maintain healthy sleep behaviors, making them more prone to anxiety and helplessness before sleep, which in turn impairs sleep quality and exacerbates sleep-related worry. In shift nurses with “Consequence Worry,” persistent worry about the consequences of sleep consumes substantial psychological resources, leaving them with insufficient self-regulatory capacity. When self-regulatory fatigue accumulates to a threshold, nurses find it difficult to manage sleep-related worry and the negative emotions and work engagement problems it triggers, eventually evolving into the “Generalized Dysregulation” sleep-related worry pattern.

Presenteeism is a behavioral marker unique to the “Generalized Dysregulation” sleep-related worry group. Nurses in the “Consequence Worry” group were characterized primarily by anticipatory anxiety; although their cognitive resources were under high demand, this had not yet fully translated into behavioral dysfunction. In contrast, for nurses in the “Generalized Dysregulation” group, their worry had already crossed the cognitive boundary and permeated actual work performance, with attention, memory, and executive function severely compromised as cognitive resources were occupied by persistent worry. Nursing managers are unlikely to identify this subgroup through routine indicators such as attendance rates. Presenteeism has a negative impact on nursing quality, as nurses whose cognitive resources are occupied by sleep-related worry show significant declines in reaction speed and judgment accuracy when performing complex procedures, making clinical decisions, or responding to emergencies (41). This increases the risk of adverse events and compromises both patient safety and quality of care. Baek et al. (42) also confirmed that adequate sleep can effectively reduce presenteeism among shift nurses. The SPS-6 can serve as a simple screening tool for identifying high-risk nurses in the “Generalized Dysregulation” group, thereby filling the blind spots left by routine indicators such as attendance rates.

Under the current performance-based compensation system, shift nurses’ pay is directly tied to the frequency of night shifts and total working hours. Consequently, a moderate income level is often accompanied by a higher number of night shifts and longer work hours. When shift nurses trade sleep for income growth, the health costs continue to rise with cumulative night shifts. Nurses with a moderate income are often at a critical stage of career development, bearing heavy clinical and teaching responsibilities while lacking scheduling autonomy. Facing a structural dilemma of “high input, moderate reward,” they are susceptible to chronic stress and emotional exhaustion, which in turn exacerbates the generalization of sleep-related worry (43). The risk of being classified into the “Generalized Dysregulation” group tended to diminish among nurses with a monthly income above 10,000 RMB, which may be attributable to their greater scheduling autonomy or opportunities for position adjustment, thereby attenuating the positive association between income and sleep-related worry.

Shift nurses who frequently participated in sleep-related training showed a protective effect against the “Consequence Worry” sleep-related worry group. Shift nurses who “frequently” participated in training acquired comprehensive sleep knowledge and coping strategies through continuous systematic learning, enabling them to effectively manage sleep-related worry under the pressure of shift work. Shift nurses who “occasionally” participated in training were unable to engage systematically or consistently in sleep-related training due to various reasons, such as shift conflicts, insufficient training resources, and limited personal time. The fragmented knowledge they acquired failed to provide effective coping tools, and may have paradoxically intensified their worry and sense of loss of control by increasing excessive awareness of sleep problems. A systematic review has indicated that effective sleep interventions should incorporate cognitive-behavioral components, rather than relying solely on didactic knowledge transfer (44). Sleep health training should achieve full coverage through institutionalized arrangements at the organizational level, so as to avoid the dilemma of relying on voluntary participation—namely, that low-risk individuals do not attend, while high-risk individuals derive limited benefit from participation. Shift nurses who frequently participated in sleep-related training showed a protective effect against the “Consequence Worry” sleep-related worry group.

As an acute stressor, occasional overtime primarily disrupts the sleep preparation process and triggers anticipatory worry about work-related consequences, as nurses can still maintain a dynamic balance of resources through intermittent recovery windows. Frequent overtime, by continuously compressing recovery time, keeps shift nurses in a sustained state of resource depletion, ultimately leading to generalized dysregulation of sleep-related worry. Studies have shown that higher frequency of night shifts, extended work hours, and insufficient rest time are all associated with increased levels of occupational stress and anxiety (45). When overtime becomes routine, shift nurses’ recovery time is systematically compressed, leaving their psychological resources in a prolonged state of negative balance, which further exacerbates sleep-related worry.

### 4.3 Intervention strategies

For shift nurses in the C3 group, nursing managers should appropriately reduce the weight of night shift frequency in performance-based compensation allocation, so as to prevent nurses from passively taking on excessive night shifts in pursuit of higher income. Performance evaluation should incorporate a nursing quality coefficient (e.g., quality assessment, incidence of adverse events, patient satisfaction) and a critical care difficulty coefficient (with differentiated weights based on disease severity and procedural complexity), so that performance rewards more adequately reflect nursing quality and professional value, rather than relying solely on the number of night shifts (46). In addition, information technology should be used to achieve transparent performance management, ensuring that compensation allocation favors frontline positions with high difficulty, high risk, and high skill requirements. It is recommended that hospitals establish a strict cap of no more than three overtime shifts per month, and introduce an AI-powered shift scheduling system with functions for automatic work-hour monitoring and rule-violation blocking (47, 48). When a proposed schedule approaches or exceeds the overtime limit, the system would automatically issue alerts and prevent the schedule from being finalized, thereby safeguarding nurses’ work-life balance. The development of system rules should be jointly negotiated by the nursing department, human resources department, and labor union to ensure fairness and enforceability. For departments where nurses exceed the overtime limit more than three times per month, it is recommended to establish a nurse manager accountability mechanism, using a progressive model of “diagnosis–support–rectification.” Following a joint analysis by the nursing department and the human resources department, targeted resource support should be provided: for departments with recurrent violations due to systematic understaffing, a hospital-wide human resource allocation mechanism should be activated; for recurrent violations attributable to mismanagement, the issue should be incorporated into the nurse manager’s performance evaluation. Accountability is intended to drive systemic improvement, rather than to personalize organizational problems. In this study, 71.4% of married nurses were classified into the C3 group, with the dual burden of family caregiving responsibilities and clinical work being an important driver (49). It is recommended that hospital trade unions, in collaboration with relevant departments, provide family caregiving support, such as issuing emergency care vouchers, and actively respond to the national policy direction of “encouraging employers to provide childcare services”. This would help alleviate the pressure on nurses who are forced to forego rest or attend work while ill due to sudden family emergencies (50). It is recommended that shift nurses within the same group establish peer support partnerships, regularly exchange sleep improvement experiences, and thereby reduce the shame and isolation associated with sleep problems, while gaining practical advice and emotional support.

It is recommended that nurses in the C2 group be designated as a “high-risk conversion population” and be prioritized for inclusion in hospital mental health promotion programs. At the psychological support level, the occupational health department should provide unified access to mindfulness-based stress reduction (MBSR) training (51) and an expedited pathway to psychological counseling, so as to prevent their transition to the C3 group. At the organizational scheduling level, managers should be authorized to implement pilot scheduling optimization measures in departments with a high concentration of C2 group nurses, such as reducing rapid shift rotations and enforcing mandatory rest days after night shifts (52). The effectiveness of these measures should be evaluated by the Nursing Quality Management Committee, with successful practices scaled up accordingly. Any additional labor costs incurred during the pilot phase should be considered a preventive investment. At the level of dynamic monitoring, it is recommended that the ASPQ be administered quarterly for re-assessment. For nurses whose total scores show a persistent upward trend, additional psychological support resources should be proactively provided. The nursing department should organize group education on sleep restriction therapy for nurses in the C2 group (53). Based on sleep diary records, average sleep duration should be calculated and an initial time-in-bed window should be set. As sleep efficiency improves (≥85%), the time-in-bed window should be gradually expanded. By reducing the amount of time spent awake in bed, this approach may help compress the cognitive space for sleep-related worry. Nurses should perform 3–5 min of mindful breathing exercises daily, using mindfulness mobile apps or MBSR recordings provided by the hospital to assist their practice. Sleep hygiene practices (20) should include avoiding caffeine intake within 4 h before bedtime, refraining from discussing work-related stress before sleep, and ensuring that the bedroom is completely dark and quiet after night shifts.

Nurses in the C1 group should be identified as departmental “sleep health role models.” They should be encouraged to volunteer as sleep health facilitators, providing peer support to nurses in other groups and helping C2 and C3 group nurses develop reasonable expectations about sleep through experience sharing (54). The nursing department and human resources department should jointly provide training for sleep health facilitators. The training content should include: foundational knowledge of sleep science, scientific principles and practical strategies for shift work adaptation, peer support communication skills, and stress management and mindfulness basics. The training should be arranged during working hours or counted as continuing education credits, so as to avoid encroaching on nurses’ rest time. A special fund for a “healthy attendance” organizational culture should be established to support the equipment and maintenance of night-shift rest rooms, as well as the provision of recovery resources such as hot beverages and light snacks. Funding should be sourced from the hospital’s occupational health budget, union funds, or external project grants, to ensure that C1 group nurses who serve as sleep health facilitators receive tangible organizational recognition and financial compensation for their role.

### 4.4 Limitations

This study has several limitations. First, its cross-sectional design does not allow for causal inferences. Future longitudinal or interventional studies are warranted to further examine the developmental trajectories of sleep-related worry among shift nurses and their implications for sleep health. Second, this study was conducted in a single region, and the geographical limitation of the sample may restrict the generalizability of the findings to other regions, different types of hospitals, or nurse populations with varying demographic characteristics. Therefore, future research should focus on expanding sample diversity. On the one hand, nurses from multiple geographic regions should be included to verify whether the three-class structure of sleep-related worry is replicable across different cultural and healthcare system contexts. On the other hand, nurses from hospitals of different levels and types should be recruited to assess whether the distribution patterns and influencing factors of sleep-related worry vary according to organizational characteristics. Finally, all data in this study were collected through self-reports from shift nurses, which may introduce information bias. Future research could incorporate objective sleep monitoring data (e.g., actigraphy, polysomnography) and other multi-source data to reduce the potential influence of self-report bias on the findings.

## 5 Summary

The present study found that sleep-related worry among shift nurses exhibits significant group heterogeneity and can be classified into three latent classes: “Generalized Dysregulation” (58.35%), “Consequence Worry” (25.11%), and “Emotionally Healthy” (16.54%). Notably, 83.46% of the nurses fell into the moderate- to high-risk classes, indicating that sleep-related worry is a widespread occupational health issue among shift nurses. The “Generalized Dysregulation” class was characterized by multidimensional functional impairments, including severely compromised sleep quality, marked depletion of self-regulatory capacity, and substantial decline in work efficiency. The “Consequence Worry” class represented a transitional state characterized by high cognitive resource consumption, without yet fully manifesting overt behavioral dysfunction. The “Emotionally Healthy” class was a low-risk group with relatively good psychological resource reserves, exhibiting only situational worry following high-intensity work exposure. Sleep quality, self-regulatory fatigue, presenteeism, monthly income, frequency of sleep-related training, and overtime frequency were important influencing factors of different latent classes of sleep-related worry among shift nurses. Accordingly, we recommend implementing tiered, targeted interventions. For the “Generalized Dysregulation” class, a systematic multidimensional approach should be adopted, including digital CBT-I, performance-based salary reform, a strict overtime cap with an AI-powered scheduling system, and family caregiving support for married nurses. For the “Consequence Worry” class, during the window period of potential progression to the “Generalized Dysregulation” class, interventions such as cognitive restructuring, mindfulness-based stress reduction, and sleep restriction therapy should be prioritized, alongside optimized shift scheduling. For the “Emotionally Healthy” class, the focus should be on health promotion and positive reinforcement, encouraging these nurses to serve as departmental sleep health facilitators and provide peer support.

## Data Availability

The original contributions presented in the study are included in the article/Supplementary material, further inquiries can be directed to the corresponding author.

## References

[1] Tang NKY Harvey AG. Correcting distorted perception of sleep in insomnia: a novel behavioural experiment? Behav Res Ther. (2004) 42:27–39. doi: 10.1016/S0005-7967(03)00068-8, 14744521

[2] Jansson-Fröjmark M Harvey A Lundh L-G Norell A Linton S. Psychometric properties of an insomnia-specific measure of worry: the anxiety and preoccupation about sleep questionnaire. Cogn Behav Ther. (2011) 40:65–76. doi: 10.1080/16506073.2010.538432, 21337216

[3] Riemann D Spiegelhalder K Feige B Voderholzer U Berger M Perlis M . The hyperarousal model of insomnia: a review of the concept and its evidence. Sleep Med Rev. (2010) 14:19–31. doi: 10.1016/j.smrv.2009.04.002, 19481481

[4] Simonetti V Durante A Ambrosca R Arcadi P Graziano G Pucciarelli G . Anxiety, sleep disorders and self-efficacy among nurses during COVID-19 pandemic: a large cross-sectional study. J Clin Nurs. (2021) 30:1360–71. doi: 10.1111/jocn.15685, 33534934 PMC8012992

[5] Aemmi SZ Mohammadi E Fereidooni-Moghadam M Zarea K Boostani H. Sleep management experiences of shift-working nurses: a grounded theory study. Collegian. (2022) 29:493–9. doi: 10.1016/j.colegn.2021.11.005

[6] Lim H Kim SH. Navigating night shifts: a qualitative study of exploring sleep experiences and coping strategies among nurses. BMC Nurs. (2025) 24:385. doi: 10.1186/s12912-025-03001-3, 40197250 PMC11978025

[7] Feng G Qiping A. Status quo and influencing factors of sleep concerns among shift nurses in emergency department. Chin Nurs Res. (2024) 38:3745–9. doi: 10.12102/j.issn.1009-6493.2024.20.033

[8] Yang C Cheng J Xiao L Tian J Zhang D Ren J. Current status and influencing factors of sleep-related worry among nurses in the department of anesthesiology: a cross-sectional study. BMC Nurs. (2025) 24:197. doi: 10.1186/s12912-025-02833-3, 39979964 PMC11844091

[9] Hittle BM Hils J Fendinger SL Wong IS. A scoping review of sleep education and training for nurses. Int J Nurs Stud. (2023) 142:104468. doi: 10.1016/j.ijnurstu.2023.104468, 37080122 PMC10180237

[10] Xu S Ouyang X Shi X. Emotional exhaustion and sleep-related worry as serial mediators between sleep disturbance and depressive symptoms in student nurses: a longitudinal analysis. Chin J Clin Psychol. (2023) 31:1180–3. doi: 10.16128/j.cnki.1005-3611.2023.05.030 31862630

[11] Li Y Wang Y Lv X Li R Guan X Li L . Effects of factors related to shift work on depression and anxiety in nurses (2022) 10:2022. Front. Public Health, English. doi: 10.3389/fpubh.2022.926988 PMC9326492 35910870

[12] Vestergaard JM Dalbøge A Bonde JPE Garde AH Hansen J Hansen ÅM . Night shift work characteristics and risk of incident coronary heart disease among health care workers: national cohort study. Int J Epidemiol. (2023) 52:1853–61. doi: 10.1093/ije/dyad126, 37741924 PMC13030994

[13] James L James SM Wilson M Brown N Dotson EJ Dan Edwards C . Sleep health and predicted cognitive effectiveness of nurses working 12-hour shifts: an observational study. Int J Nurs Stud. (2020) 112:103667. doi: 10.1016/j.ijnurstu.2020.103667, 32593476

[14] Lin L Gao Z Peng Y Li S Chen L Lin Y. The relationship between poor sleep and memory impairment among shift nurses in China: a cross-sectional study. Nat Sci Sleep. (2024) 16:1653–63. doi: 10.2147/nss.S474113, 39440275 PMC11495186

[15] Park E Lee HY Park CS-Y. Association between sleep quality and nurse productivity among Korean clinical nurses. J Nurs Manag. (2018) 26:1051–8. doi: 10.1111/jonm.12634, 29855101

[16] Stimpfel AW Fatehi F Kovner C. Nurses' sleep, work hours, and patient care quality, and safety. Sleep Health. (2020) 6:314–20. doi: 10.1016/j.sleh.2019.11.001, 31838021

[17] Steven A Redfern N. Fatigue: for safe patients we need safe nurses. J Adv Nurs. (2025) 81:5627–31. doi: 10.1111/jan.16231, 38733077 PMC12371789

[18] Li Y Zhang R Kang L Li J Yi Z Zhao H . Relationship between sleep-related worry, work well-being, and retention intention among nurses: a cross-sectional multicenter study. J Nurs Manag. (2025) 2025:8839164. doi: 10.1155/jonm/8839164, 41048833 PMC12494469

[19] Ki J Ryu J Baek J Huh I Choi-Kwon S. Association between health problems and turnover intention in shift work nurses: health problem clustering. Int J Environ Res Public Health. (2020) 17:1–12. doi: 10.3390/ijerph17124532, 32599700 PMC7345885

[20] Sun Q Ji X Zhou W Liu J. Sleep problems in shift nurses: a brief review and recommendations at both individual and institutional levels. J Nurs Manag. (2019) 27:10–8. doi: 10.1111/jonm.12656, 30171641

[21] Kagamiyama H Sumi N Yoshida Y Sugimura N Nemoto F Yano R. Association between sleep and fatigue in nurses who are engaged in 16 h night shifts in Japan: assessment using actigraphy. Jpn J Nurs Sci. (2019) 16:373–84. doi: 10.1111/jjns.12246, 30585410

[22] Harvey AG. A cognitive model of insomnia. Behav Res Ther. (2002) 40:869–93. doi: 10.1016/S0005-7967(01)00061-4 12186352

[23] Silva-Costa A Ferreira PCS Griep RH Rotenberg L. Association between Presenteeism, psychosocial aspects of work and common mental disorders among nursing personnel. Int J Environ Res Public Health. (2020) 17:1–18. doi: 10.3390/ijerph17186758, 32948065 PMC7559752

[24] Wu J Li Y Lin Q Zhang J Liu Z Liu X . The effect of occupational coping self-efficacy on presenteeism among ICU nurses in Chinese public hospitals: a cross-sectional study. Front Psychol. (2024) 15:1347249. doi: 10.3389/fpsyg.2024.1347249, 38356774 PMC10865889

[25] Zeng Q Zhang Y Liang H Li Y Zhang X Wang C . The mediating role of self-regulatory fatigue on the relationship between insomnia and sleep-related worry among shift nurses: a cross-sectional study. BMC Nurs. (2025) 25:105. doi: 10.1186/s12912-025-04258-4, 41469655 PMC12859850

[26] Ji Y Min A Kang M Park C. How shift nurses' Presenteeism is related to insomnia and care left undone: a cross-sectional study using generalised structural equation modelling. J Adv Nurs. (2025) 81:3692–702. doi: 10.1111/jan.16483, 39315744

[27] Rosa D Terzoni S Dellafiore F Destrebecq A. Systematic review of shift work and nurses’ health. Occup Med (Lond). (2019) 69:237–243. doi: 10.1093/occmed/kqz063 31132107

[28] Nylund-Gibson K Garber AC Carter DB Chan M Arch DAN Simon O . Ten frequently asked questions about latent transition analysis. Psychol Methods. (2023) 28:284–300. doi: 10.1037/met0000486, 35834194

[29] Preacher KJ Kelley K. Effect size measures for mediation models: quantitative strategies for communicating indirect effects. Psychol Methods. (2011) 16:93–115. doi: 10.1037/a0022658, 21500915

[30] Nes LS Ehlers SL Whipple MO Vincent A. Self-regulatory fatigue in chronic multisymptom illnesses: scale development, fatigue, and self-control. J Pain Res. (2013) 6:181–8. doi: 10.2147/jpr.S40014, 23526193 PMC3596127

[31] Ligang W Jingyi Z Jia W. Validity and reliability of the Chinese version of the self-regulatory fatigue scale in young adults. Chin Ment Health J. (2015) 29:290–4. doi: 10.3969/j.issn.1000-6729.2015.04.010

[32] Buysse DJ Reynolds CF 3rd Monk TH Berman SR Kupfer DJ. The Pittsburgh sleep quality index: a new instrument for psychiatric practice and research. Psychiatry Res. (1989) 28:193–213. 2748771 10.1016/0165-1781(89)90047-4

[33] Koopman C Pelletier KR Murray JF Sharda CE Berger ML Turpin RS . Stanford presenteeism scale: health status and employee productivity. J Occup Environ Med. (2002) 44:14–20. doi: 10.1097/00043764-200201000-00004, 11802460

[34] Fang Z Junming D Shiyong Y. Reliability and validity of the Chinese version of the Stanford Presenteeism scale (SPS-6). Chin J Ind Hyg Occup Dis. (2010) 28:679–82. doi: 10.3760/cma.j.issn.1001-9391.2010.09.012 21126483

[35] Booker LA Barnes M Alvaro P Collins A Chai-Coetzer CL McMahon M . The role of sleep hygiene in the risk of shift work disorder in nurses. Sleep. (2020) 43:zsz228. doi: 10.1093/sleep/zsz228, 31637435

[36] Moosavi S Ghalenoei M Amerzadeh M Variani AS. The relationship between shift work, circadian rhythms, and cognitive function in ICU nursing. BMC Nurs. (2025) 24:324. doi: 10.1186/s12912-025-02850-2, 40140864 PMC11948777

[37] Hiller RM Johnston A Dohnt H Lovato N Gradisar M. Assessing cognitive processes related to insomnia: a review and measurement guide for Harvey's cognitive model for the maintenance of insomnia. Sleep Med Rev. (2015) 23:46–53. doi: 10.1016/j.smrv.2014.11.006, 25645129

[38] Xu S Ouyang X Shi X Li Y Chen D Lai Y . Emotional exhaustion and sleep-related worry as serial mediators between sleep disturbance and depressive symptoms in student nurses: a longitudinal analysis. J Psychosom Res. (2020) 129:109870. doi: 10.1016/j.jpsychores.2019.109870, 31862630

[39] Law C Sokolovsky AW Yap DL Erblich J Gunthert K Behar E. Examining the reciprocal association between worry and sleep: disaggregating between- and within-person effects. Behav Ther. (2025) 56:634–47. doi: 10.1016/j.beth.2024.09.005, 40287189

[40] Liu Y Zhao X. The impact of emotional labor on sleep-related worry among pediatric nurses in Chinese public hospitals: the mediating role of self-regulatory fatigue (2026) 14:1–14. doi: 10.3389/fpubh.2026.1837829, PMC13233409 42254626

[41] Hou H Jiang Y Xia C Cheng X Li H Dai H . The lived experience of presenteeism among emergency nurses: a qualitative study. Front. Public Health. (2025). 24:1255. doi: 10.1186/s12912-025-03902-3, 41068708 PMC12512700

[42] Baek J Ki J Ryu J Smi C-K. Relationship between occupational stress, sleep disturbance, and presenteeism of shiftwork nurses. J Nurs Scholarsh. (2022) 54:631–8. doi: 10.1111/jnu.12766, 35084088

[43] Lv F Li L Wang N . Impact of effort‑reward imbalance, emotional labour, and nurse‑patient relationship on physical and mental health of registered nurses in China: a structural equation modeling. BMC Nurs. (2025) 24:535. doi: 10.1186/s12912-025-03147-0 40369545 PMC12080039

[44] Zhang Y Murphy J Lammers-van der Holst HM Barger LK Lai YJ Duffy JF. Interventions to improve the sleep of nurses: a systematic review. Res Nurs Health. (2023) 46:462–84. doi: 10.1002/nur.22337, 37710916 PMC10539041

[45] Yuan M-Z Fang QJJoan Latent class Analysis of the sleep Quality of night Shift Nurses and impact of Shift-Related Factors on the Occupational Stress and Anxiety Hoboken, New Jersey: John Wiley & Sons, Inc. (2024). 10.1111/jan.16067 38235926

[46] He W Du L Zhang W. Development of best performance evaluation indicators based on value-based healthcare for general hospital nursing: a Delphi study. J Clin Nurs. (2025) 34:816–25. doi: 10.1111/jocn.17405, 39152561

[47] Shia BC Peng SM Zhang QY Lo CY Wang SR. Explainable AI for equitable nurse scheduling: pragmatic pre-post implementation study. JMIR Nurs. (2026) 9:e94450. doi: 10.2196/94450, 42391503 PMC13328950

[48] Kang HW Kim J Kim KJ Bae EK Kang H Jang JH . Shift nurses’ work quality and job satisfaction after implementing the Inha University hospital nursing AI scheduling system (IH-NASS). BMC Nurs. (2025) 24:792. doi: 10.1186/s12912-025-03470-6, 40597282 PMC12210576

[49] Hwang E Yu Y. Effect of sleep quality and depression on married female nurses' work-family conflict. Int J Environ Res Public Health. (2021) 18:1–14. doi: 10.3390/ijerph18157838, 34360128 PMC8435216

[50] Watson AL Nelson B Houston G. Parenting stress and nurse workforce sustainability: an integrative literature review. J Nurs Scholarsh. (2026) 58:e70104. doi: 10.1111/jnu.70104, 42298827

[51] Dou J Lian Y Lin L Asmuri SNB Wang P Rajen Durai RAP. Effectiveness of mindfulness-based interventions on burnout, resilience and sleep quality among nurses: a systematic review and meta-analysis of randomized controlled trials. BMC Nurs. (2025) 24:739. doi: 10.1186/s12912-025-03101-0, 40598019 PMC12210539

[52] Kim SH Jang G Lee Y Lee S Hwang H Shin S . Data-driven scheduling strategies to minimize fatigue in rotating night-shift nurses. J Nurs Manag. (2026) 2026:e9994492. doi: 10.1155/jonm/9994492, 42478069 PMC13385972

[53] Looman MI Klau MM Schoenmakers TM Kamphuis JH Blanken TF Lancee J. The adherence conundrum of sleep restriction therapy for insomnia. J Sleep Res. (2026) 35:e70339. doi: 10.1111/jsr.70339, 41918497 PMC13357881

[54] Nilsson O Vehkala-Höglund A Gellerstedt L. Evidence-based sleep promotion in acute care from the perspective of nursing staff: a cross-sectional study. BMC Nurs. (2026) 25:32. doi: 10.1186/s12912-025-04259-3, 41491189 PMC12797770

