# Supplementary material for: Latent class analysis of sleep-related worry and its influencing factors among shift work nurses: a cross-sectional study
Source: Front Public Health. 2026 Sep 3;14:1908778. doi: 10.3389/fpubh.2026.1908778 (PMC13581642; doi:10.3389/fpubh.2026.1908778)
Supplement: Supplementary file 1 [file Table_1.pdf]

Supplementary Material

1 Supplementary Data

Table 4. Multinomial logistic regression analysis of latent classes of sleep-related worry among shift nurses (n = 653)

Variables	C2 vs C1					C3 vs C1				
	β	*SE*	*Waldχ²*	*P*	*OR (95% CI)*	*β*	*SE*	*Waldχ²*	*P*	*OR (95% CI)*
Marital status										
Unmarried	0.309	0.567	0.297	0.586	1.362(0.448-4.143)	0.184	0.740	0.062	0.804	1.202(0.282-5.122)
self-rated sleep quality										
Good sleep quality	0.994	0.757	1.727	0.189	2.703(0.613-11.913)	-1.949	1.394	1.955	0.162	0.142(0.009-2.188)
self-rated health status										
Good self-rated health	-1.936	1.346	2.069	0.150	0.144(0.010-2.018)	0.132	0.619	0.045	0.832	1.141(0.339-3.837)
Years of work experience										
0-1(years)	-0.628	1.046	0.360	0.549	0.534(0.069-4.149)	-1.778	1.223	2.111	0.146	0.169(0.015-1.859)
2-5(years)	0.065	0.732	0.008	0.929	1.067(0.254-4.483)	-0.296	0.841	0.124	0.725	0.744(0.143-3.863)

6-10(years)	-0.798	0.601	1.763	0.184	0.450(0.139-1.462)	-0.759	0.696	1.188	0.276	0.468(0.120-1.833)
11-15(years)	-0.831	0.649	1.637	0.201	0.436(0.122-1.566)	-0.594	0.746	0.633	0.426	0.552(0.128-2.384)
Monthly income (RMB)										
＜4000	0.624	1.118	0.311	0.577	1.866(0.209-16.696)	2.009	1.325	2.297	0.130	7.454(0.555-100.112)
4000-6000	0.654	0.691	0.896	0.344	1.923(0.496-7.451)	1.244	0.780	2.541	0.111	3.468(0.752-16.004)
6001-8000	0.899	0.564	2.539	0.111	2.457(0.813-7.420)	1.905	0.620	9.440	0.002*	6.717(1.993-22.639)
8001-10000	0.641	0.467	1.884	0.170	1.897(0.760-4.735)	1.001	0.517	3.748	0.053	2.72(0.988-7.493)
sleep-related training										
frequently	-2.005	1.017	3.886	0.049*	0.135(0.018-0.988)	-1.718	1.233	1.941	0.164	0.179(0.016-2.011)
Sometimes	-0.625	0.720	0.753	0.385	0.535(0.131-2.195)	0.455	0.806	0.319	0.572	1.577(0.325-73659)
Occasionally	0.819	0.677	1.463	0.226	2.267(0.602-8.543)	1.680	0.728	5.319	0.021*	5.363(1.287-22353)
mental health training										
frequently	-1.190	0.910	1.708	0.191	0.304(0.051-1.812)	-1.239	1.082	1.311	0.252	0.290(0.035-2.416)
Sometimes	-0.019	0.721	0.001	0.979	0.981(0.239-4.030)	-0.614	0.785	0.613	0.434	0.541(0.116-2.518)
Occasionally	-0.120	0.467	0.066	0.797	0.887(0.355-2.214)	-0.433	0.526	0.679	0.410	0.648(0.231-1.817)

shift length										
12-hour shift	-0.022	0.572	0.002	0.969	0.978(0.319-3.001)	0.171	1.198	0.020	0.886	1.187(0.113-12.424)
shift pattern										
Two-shift system	0.185	1.128	0.027	0.870	1.203(0.132-10.983)	-0.265	1.171	0.051	0.821	0.767(0.077-7.618)
Three-shift system	-0.518	1.104	0.220	0.639	0.596(0.069-5.181)	0.474	0.625	0.574	0.449	1.606(0.471-5.471)
frequency of working more than 40 hours per month										
1	0.978	0.487	4.036	0.045*	2.659(1.024-6.902)	0.937	0.529	3.134	0.077	2.551(0.905-7.197)
2	0.412	0.529	0.608	0.435	1.510(0.536-4.257)	0.217	0.570	0.144	0.704	1.242(0.406-3.795)
3	0.389	0.578	0.454	0.500	1.476(0.476-4.579)	1.314	0.622	4.469	0.035*	3.722(1.101-12.591)
sleep quality	0.457	0.076	36.256	＜0.001*	1.579(1.361-1.832)	0.904	0.088	105.248	＜0.001*	2.470 (2.078-2.936)
self-regulatory fatigue	0.081	0.029	7.487	0.006*	1.084(1.023-1.148)	0.136	0.032	18.305	＜0.001*	1.145(1.076-1.219)
presenteeism	0.048	0.047	1.057	0.304	1.049(0.957-1.150)	0.108	0.051	4.479	0.034*	1.114(1.008-1.232)

Note: C1, emotionally healthy sleep-related worry group; C2, consequence worry sleep-related worry group; C3, generalized dysregulation sleep-related worry group. *，The difference was statistically significant ($P<0.05$).
